# Associations of perceived patient safety culture and work-related characteristics with self-reported shared governance capacity among nurses in a tertiary hospital in China: a cross-sectional study

**DOI:** 10.3389/fmed.2026.1897058

**Published:** 2026-08-19

**Authors:** Xingxing Xu, Ling Zhang, Xueying Shao, Qian Zhang, Haifang Zhou

**Affiliations:** 1 Department of TCM Nursing Clinic, Hangzhou TCM Hospital Affiliated to Zhejiang Chinese Medical University, Hangzhou, China; 2 Department of Cardiovascular Medicine, Maoming People's Hospital, Maoming, China; 3 Department of Nursing, Hangzhou TCM Hospital Affiliated to Zhejiang Chinese Medical University, Hangzhou, China

**Keywords:** decision-making, hospital management, nurse, patient safety culture, shared governance, work-related characteristics

## Abstract

**Purpose:**

This cross-sectional study aimed to assess nurses’ shared governance capacity in a tertiary hospital in China and examine its associations with perceived patient safety culture, demographic characteristics, and work-related characteristics.

**Methods:**

A total of 755 nurses from a tertiary general hospital in China completed a confidential online survey using the Shared Governance Capacity Scale for Nurses and the Hospital Survey on Patient Safety Culture. Hierarchical multiple linear regression analysis was conducted to examine factors associated with nurses’ shared governance capacity, with shared governance capacity as the dependent variable and perceived patient safety culture, demographic characteristics, and work-related characteristics as independent variables. The study adhered to the Strengthening the Reporting of Observational Studies in Epidemiology (STROBE) guidelines for observational studies.

**Results:**

Overall, 61.1% of nurses did not meet the predefined adequacy criterion of the Shared Governance Capacity Scale for Nurses, defined as a score of ≥36, and the mean shared governance capacity score was relatively low (35.46 ± 6.72). Hierarchical multiple linear regression analysis showed that higher perceived patient safety culture and having more than 5 years of work experience were associated with higher shared governance capacity, whereas unmarried status, working overtime, and poor sleep quality were associated with lower shared governance capacity.

**Conclusion:**

Nurses’ shared governance capacity was relatively low in this tertiary hospital in China and was associated with perceived patient safety culture, work experience, working overtime, marital status, and sleep quality. These findings may inform future efforts to create organizational conditions that support nurses’ participation in shared governance.

## 1 Introduction

Shared governance is an organizational model grounded in the principles of professional empowerment and participatory decision-making. This model emphasizes collaborative decision-making between nurses and managers in nursing administration (1). Shared governance, as recognized by the American Nurses Credentialing Center (ANCC), is a cornerstone of the Magnet Hospital Model and serves as a benchmark for high-performing nursing organizations (2). Research has demonstrated that shared governance is associated with nurses’ sense of organizational belonging, autonomy, and responsibility, as well as care quality, improving patient outcomes, and increasing employee satisfaction (3, 4).

In China, however, the role of nurses in organizational decision-making is often marginalized in some hospital contexts due to the traditional hierarchical management system and limited nursing workforce resources. In contrast, in developed countries such as the United States (U.S.), shared governance has shown considerable success. A study conducted in 27 U.S. hospitals revealed that shared governance restored meaningful recognition for nurses, positively impacting nurses, patients, and the healthcare system (4). For instance, shared governance contributed to a reduction in the number of patient falls, the development of patient education materials, and improved nurse retention (5). Hospitals that adopted shared governance models reported significant improvements in nurse satisfaction, lower turnover rates, and a 22% decrease in safety events (3). Additionally, countries such as Finland and other Nordic nations have incorporated shared governance as a key element of hospital governance reform, fostering substantial nurse participation in areas such as scheduling, material management, and quality improvement through nursing committees and shared decision-making platforms (6). Shared governance is a key evaluation criterion for Magnet® hospitals. According to the latest data from the ANCC, there are 629 Magnet-recognized hospitals, accounting for approximately 10% of all hospitals nationwide in the US. However, the adoption of shared governance models likely extends well beyond Magnet institutions themselves (7). These findings suggest the potential value of shared governance. However, the studies cited above were conducted primarily in the United States and Nordic healthcare systems, where governance structures and nursing workforce resources may differ from those in China.

In contrast, in China, shared governance remains relatively underdeveloped, hindered by an underdeveloped institutional foundation and an insufficient cultural atmosphere. China had 4.0 RNs per 1,000 population by the end of 2023, according to official data released in 2024 (8). China’s nurse density remained below the estimated threshold of 7.06 RNs per 1,000 people associated with universal health coverage performance in the GBD study (9). Furthermore, traditional hierarchical management remains common in most Chinese hospitals, leaving nurses with limited decision-making power and few opportunities to engage in hospital governance. This situation may undermine career development and organizational identity among nurses (10). Nurses often find themselves in passive, executive roles compounded by the high work intensity, slow pace of promotions, and significant assessment-related pressure. As a result, the development of nurses’ governance capacity may be hindered, limiting improvements in organizational efficiency and governance quality (11).

Enhancing nurses’ shared governance capacity has become a critical issue in the context of China’s strategic focus on the ‘high-quality development of healthcare’. Nurses, who account for over 59% of healthcare providers (12), play a pivotal role in patient care and are at the frontline of healthcare delivery. The involvement of nurses in decision-making directly influences patient safety, care quality, and organizational responsiveness (13). Patient safety culture reflects organizational support for safe care, including teamwork, communication, staffing, and management support for patient safety (14). Studies have shown that shared governance capacity is not only linked to nurses’ self-efficacy and sense of responsibility but is also strongly associated with the teamwork atmosphere, communication efficiency, and risk management awareness (3). However, domestic research on nurses’ shared governance capacity and its associated factors remains limited. Evidence is particularly scarce in Chinese hospital settings with constrained nursing workforce resources, where nurses face high workload, limited staffing support, and restricted participation in organizational decision-making (15). Although recent studies have examined shared governance in relation to professional governance interventions, autonomy, nurse satisfaction, and nurse-sensitive outcomes, its direct associations with perceived patient safety culture and specific work-related characteristics have received limited attention (16, 17).

The person–environment fit model suggests that employee behavioural competencies result from interactions between individual characteristics and the organizational environment (18). This model provided a theoretical basis for examining both individual characteristics and work-related organizational conditions. Therefore, in this study, nurses’ shared governance capacity was considered a composite skill related to both personal and professional background factors, such as career development stage, gender, age, years of experience, and education level (19). It was also considered to be related to organizational factors, such as work environment risks, resource support, work overtime, night-shift burden, and perceived culture of patient safety at the hospital (20). In the present study, this framework was used to examine workforce characteristics, patient safety culture, and shared governance capacity within the same analytical model.

In summary, enhancing nurses’ shared governance may not only enhance their professional satisfaction, sense of belonging, and sense of purpose but is also associated with improved patient outcomes, a reduced number of medical errors, and organizational response efficiency (3, 21). In the current context of pursuing high-quality nursing services and improving hospital management performance, systematic research on nurses’ shared governance capacity has important practical significance and policy value.

Thus, this study examined nurses’ self-reported shared governance capacity in a tertiary hospital setting with constrained nursing workforce resources in China and explored its associations with perceived patient safety culture, demographic characteristics, and work-related characteristics. The findings may provide practical evidence for strengthening nursing workforce governance, patient safety culture, and organizational responsiveness in hospital settings with similar workforce and organizational characteristics.

## 2 Methods

### 2.1 Study design

This was a cross-sectional study that adhered to the Strengthening the Reporting of Observational Studies in Epidemiology guidelines for observational studies (22).

### 2.2 Participants

Clinical nurses were recruited from Maoming People’s Hospital, a tertiary hospital in a southern city, China, between December 15 and December 30, 2024. During the study period, 923 clinical nurses met the eligibility criteria. The survey invitation was sent to all of them through Questionnaire Star, a widely used online survey platform in China. A total of 841 questionnaires were submitted, giving a questionnaire submission rate of 91.1%. After excluding 86 incomplete questionnaires, 755 valid questionnaires were retained for analysis. The completion rate among submitted questionnaires was 89.8%, and the overall valid response rate among invited nurses was 81.8%. The participant recruitment and questionnaire inclusion process is shown in Supplementary Figure S1. Participation was voluntary and confidential. No direct personal identifiers were collected. The survey platform was configured to permit only one submission from each IP address. IP address information was recorded automatically by the platform solely to enforce this restriction. The raw platform record contained the IP address field, and access to this record was limited to the research team during data screening. It was not used to identify individual participants or to link questionnaire responses to individual participants. The IP address field was removed before the analytical dataset was prepared for statistical analysis. Before completing the questionnaire, participants read the study information and provided informed consent. The inclusion criteria were as follows: (1) clinical nurses who were registered nurses; and (2) nurses who understood the purpose of the study and voluntarily agreed to participate. Nurses who were currently undergoing training were excluded.

The hospital is a tertiary general hospital with approximately 2,500 beds and over 1,000 nurses, providing a robust sample base for this single-hospital study. The selected study site is located in a relatively underdeveloped region, with a GDP of approximately 407.2 billion RMB in 2024, classifying it as a typical third- or fourth-tier city (23). This setting provided a relevant context for examining nurses’ shared governance capacity in a tertiary hospital with notable workforce and organizational pressures.

Sample size estimation was performed using G*Power 3.1. An *a priori* power analysis was conducted based on linear multiple regression: fixed model, *R*^2^ increase. The input parameters were *f*^2^ = 0.02, *α* = 0.05, 1 − *β* = 0.95, number of tested predictors = 1, and total number of predictors = 13. The 13 predictors were based on the planned regression model, including perceived patient safety culture and demographic and work-related characteristics. The analysis indicated that at least 652 participants were required. Assuming a 90% response rate, the target sample size was 724 participants.

### 2.3 Measurement

#### 2.3.1 Demographic and work-related characteristics

Data on the following demographic and work-related characteristics were collected. Guided by the person-environment fit model, these characteristics were conceptually grouped into personal and professional background factors and organizational work environment factors. Personal and professional background factors included gender, age, educational level, marital status, work experience, and professional title. Organizational work environment factors included employment status, direct patient care, work overtime, clinical preceptor, night shifts per month, and sleep quality. Overtime work was assessed using a single yes-or-no item asking whether nurses usually worked beyond their scheduled working hours. Overtime frequency and duration were not assessed. Sleep quality was assessed using a single item asking nurses to rate their usual sleep quality as good or poor. Neither item used a fixed recall period and neither was derived from a validated scale. The exact item wording and response options are provided in Supplementary Table S1. Work experience was grouped as ≤5 or >5 years to distinguish nurses in the earlier stage of clinical practice from those with more established work experience. More than 10 night shifts per month indicated a relatively high night-shift burden based on routine hospital scheduling. These cutoffs were prespecified pragmatic categories rather than validated clinical thresholds. Employment status was classified as formal employment within the authorized staffing system or contract-based employment outside this system.

#### 2.3.2 Patient safety culture

Perceived patient safety culture was measured using the Hospital Survey on Patient Safety Culture (HSOPSC), developed by Sorra and Dyer (14) and adapted for Chinese contexts in 2014 by Liang (24). Liang’s Chinese validation study involved translation, back-translation, review by the original author, expert evaluation, and pilot testing. The Chinese version showed good psychometric properties, with a total Cronbach’s *α* of 0.892, a 2-week test–retest correlation of 0.854 for the total score, and a content validity index of 1.0. The scale includes 42 items rated on a 5-point Likert scale, with total scores ranging from 42 to 210 points. Higher scores indicate a better perceived patient safety culture. The validation study reported reliability estimates for the total scale, supporting the calculation and interpretation of a summed score as an overall indicator of perceived patient safety culture (24). In the present study, the total score was used because the analysis focused on overall perceived patient safety culture rather than its individual dimensions. In this study, Cronbach’s *α* was 0.923.

#### 2.3.3 Shared governance

The Shared Governance Capacity Scale for Nurses (SGCSFN), developed by Hu et al. (25), was used to assess the nurses’ shared governance capacity. Hu reported good content validity and excellent internal consistency for this instrument, with an S-CVI of 0.911 and a Cronbach’s *α* of 0.930. The scale comprises 9 items rated on a 5-point Likert scale (1 = completely noncompliant, 5 = fully compliant), yielding a total score between 9 and 45 points. A score ≥36 points was considered to indicate adequate shared governance capacity. In this study, Cronbach’s *α* was 0.935.

### 2.4 Data analysis

All statistical analyses were performed using SPSS 26.0 and R 4.4.3. Harman’s single-factor test was performed to assess potential common method bias arising from the use of self-reported questionnaires. Categorical data are expressed as frequencies and percentages, while continuous variables conforming to a normal distribution are presented as mean ± standard deviation (*M* ± SD). The normality of continuous variables was assessed using skewness and kurtosis. Pearson correlation analysis was used to examine the association between shared governance capacity and perceived patient safety culture.

Independent-samples *t* tests were used to compare shared governance capacity scores across binary demographic and work-related groups. Cohen’s d was calculated as the standardized effect size for independent-samples t tests to help interpret the practical magnitude of group differences. Hierarchical multiple linear regression analysis was conducted using the Enter method to examine factors associated with shared governance capacity. Guided by the person–environment fit model, variables were entered according to the conceptual grouping described in the Measurement section. The six personal and professional background factors were entered in Block 1, the six organizational work environment factors were added in Block 2, and perceived patient safety culture was entered in Block 3. The Enter method was applied within each block, and all 13 predictors were prespecified rather than selected according to their statistical significance in the univariate analyses. Both standardized coefficients (*β*) and unstandardized coefficients (B) with 95% confidence intervals (CIs) were generated to facilitate interpretation of the regression results. Regression assumptions were assessed before interpreting the final model. The normality of residuals was evaluated using the histogram and normal P–P plot of regression standardized residuals. Linearity and homoscedasticity were examined by inspecting the scatterplot of standardized residuals against standardized predicted values. Independence of errors was assessed using the Durbin–Watson statistic. Multicollinearity was evaluated using tolerance and variance inflation factor (VIF) values. Influential observations were assessed using Cook’s distance. Observations above the 4/n Cook’s-distance reference line were further assessed using leverage, externally studentized residuals, and DFBETAs. A one-case-deletion sensitivity analysis was also performed for the observation with the largest Cook’s distance. As a sensitivity analysis, HC3 heteroscedasticity-robust standard errors were calculated for the final regression model to assess the robustness of statistical inferences given the presence of highly imbalanced categories. A two-sided *p* value < 0.05 was considered statistically significant.

### 2.5 Ethical considerations

This study was approved by the Ethics Committee of Maoming People’s Hospital (Approval No: PJ2024MI-K144-01). All methods were performed in accordance with the relevant guidelines and regulations, including the Declaration of Helsinki. Informed consent was obtained from all participants.

## 3 Results

### 3.1 Harman’s single-factor test

Harman’s single-factor test was conducted to assess potential common method bias. The analysis extracted nine factors with eigenvalues greater than 1, and the first unrotated factor explained 32.24% of the total variance, below the 40% threshold (26). No single factor accounted for the majority of the variance. However, common method bias cannot be excluded because the principal variables were measured concurrently using self-report instruments.

### 3.2 Participant characteristics and overall shared governance capacity

The mean shared governance capacity score was 35.46 ± 6.72, and 61.1% of nurses scored below the predefined adequacy threshold of 36. Only 38.9% of nurses met the criteria for shared governance, indicating a generally low level of shared governance capacity.

A total of 755 nurses participated in the study, with a mean age of 31.99 ± 6.50 years. Most participants were female (94.2%) and aged 35 years or younger (74.6%). The majority held a bachelor’s degree or above (71.7%) and were married (68.9%). More than two-thirds had over 5 years of work experience and nearly two-thirds held junior professional titles. Most participants held contract-based positions and reported working overtime. Poor sleep quality was common, affecting 87.7% of respondents. Detailed participant characteristics are shown in Table 1.

**Table 1:** Demographic and work-related characteristics (*n* = 755).

Characteristics	Frequency (n)	Proportion (%)
Age (years)		
≤35	563	74.6
>35	192	25.4
Gender		
Male	44	5.8
Female	711	94.2
Education level		
Below bachelor’s degree	214	28.3
Bachelor’s degree and above	541	71.7
Marital status		
Married	520	68.9
Unmarried	235	31.1
Work experience (years)		
≤5	236	31.3
>5	519	68.7
Professional title		
Junior	482	63.8
Intermediate or senior	273	36.2
Employment status		
Formal employment	181	24.0
Contract-based employment	574	76.0
Direct patient care		
Yes	738	97.7
No	17	2.3
Work overtime		
Yes	726	96.2
No	29	3.8
Clinical preceptor		
Yes	244	32.3
No	511	67.7
Night shifts per month		
≤10	497	65.8
>10	258	34.2
Sleep quality		
Good	93	12.3
Poor	662	87.7

### 3.3 Correlation between shared governance and patient safety culture

The absolute values of skewness and kurtosis for shared governance capacity and perceived patient safety culture were below 2 and 7 (26), respectively, indicating that the data approximated a normal distribution. Therefore, Pearson correlation analysis was considered appropriate. The mean perceived patient safety culture score was 142.38 ± 16.36. Descriptive indices of the score distributions are shown in Table 2. Pearson correlation analysis showed that shared governance capacity was positively correlated with perceived patient safety culture (*r* = 0.391, 95% CI 0.329 to 0.450, *p* < 0.001).

**Table 2:** Correlation between shared governance capacity and perceived patient safety culture (*n* = 755).

Statistics	Shared governance	Patient safety culture
Shared governance (score)	1	
Patient safety culture (score)	0.391^***^ (0.329–0.450)	1
*M* ± SD	35.46 ± 6.72	142.38 ± 16.36
Kurtosis	−0.942	4.358
Skewness	−0.052	1.678

Values in parentheses are 95% confidence intervals for Pearson’s r. ^***^ *p* < 0.001.

### 3.4 Univariate analysis of shared governance capacity

Univariate analysis revealed that nurses who were female, were older than 35 years of age, were married, had more than 5 years of work experience, held intermediate or senior professional titles, were formally employed, and served as clinical preceptors exhibited significantly higher shared governance capacity (all *p* < 0.05). In contrast, nurses who reported working overtime, had more than 10 night shifts per month, and reported having poor sleep quality demonstrated significantly lower shared governance capacity (all *p* < 0.05). No significant associations were found between shared governance capacity and educational attainment or direct patient care responsibilities (all *p* > 0.05) (Table 3).

**Table 3:** Univariate analysis of shared governance capacity (*n* = 755).

Characteristic	Shared governance	*t-*value	*p*-value	Cohen’s *d*
Age (years)		−4.827	< 0.001	0.403
≤ 35	34.78 ± 6.80			
>35	37.45 ± 6.09			
Gender		−3.518	< 0.001	0.547
Male	32.02 ± 5.55			
Female	35.67 ± 6.73			
Education level		−1.666	0.096	0.134
Below a bachelor’s degree	34.81 ± 6.74			
Bachelor’s degree and above	35.71 ± 6.70			
Marital status		6.423	< 0.001	0.504
Married	36.48 ± 6.62			
Unmarried	33.18 ± 6.39			
Work experience (years)		−6.194	< 0.001	0.486
≤ 5	33.26 ± 6.47			
> 5	36.45 ± 6.60			
Professional title		−4.224	< 0.001	0.319
Junior	34.69 ± 6.82			
Intermediate or senior	36.81 ± 6.33			
Employment status		2.936	0.004	0.230
Formal employment	36.63 ± 5.92			
Contract-based employment	35.09 ± 6.92			
Direct patient care		−0.630	0.529	0.155
Yes	35.43 ± 6.72			
No	36.47 ± 6.84			
Work overtime		−4.094	< 0.001	0.774
Yes	35.26 ± 6.71			
No	40.41 ± 5.01			
Clinical preceptor		2.731	0.006	0.212
Yes	36.42 ± 6.22			
No	35.00 ± 6.91			
Night shifts per month		2.678	0.008	0.206
≤ 10	35.93 ± 6.64			
> 10	34.55 ± 6.79			
Sleep quality		6.140	< 0.001	0.585
Good	38.84 ± 5.51			
Poor	34.98 ± 6.74			

*t* values were calculated according to the order of categories shown in the table. Cohen’s *d* values are presented as absolute values to indicate the magnitude of the group differences.

### 3.5 Hierarchical multiple linear regression analysis of shared governance capacity

A hierarchical multiple linear regression analysis was conducted using the Enter method to examine factors associated with shared governance capacity. Predictors were entered in three blocks. The six personal and professional background factors were entered in Block 1, the six organizational work environment factors were entered in Block 2, and perceived patient safety culture was entered in Block 3, as detailed in Table 4. The assignment of dichotomous variables is detailed in Supplementary Table S2.

**Table 4:** Hierarchical multiple linear regression analysis of shared governance capacity (*n* = 755).

Panel A: model summary						
Block	Variables added	*R* ^2^	Adjusted *R*^2^	Δ*R*^2^	*F* change	*p*-value
Block 1	Personal and professional background factors	0.077	0.070	0.077	10.436	<0.001
Block 2	Organizational work environment factors	0.123	0.108	0.045	6.405	<0.001
Block 3	Perceived patient safety culture (score)	0.266	0.253	0.143	144.699	<0.001

Panel B: final model coefficients							
Independent variable	*B*	SE	*β*	*t*-value	*p*-value	95% CI	VIF
Constant	20.472	2.748		7.449	<0.001	15.077 to 25.867	
Age	0.579	0.626	0.038	0.925	0.355	−0.650 to 1.809	1.666
Gender	1.263	0.928	0.044	1.361	0.174	−0.558 to 3.085	1.058
Educational level	−0.242	0.513	−0.016	−0.471	0.638	−1.249 to 0.765	1.196
Marital status	−1.699	0.590	−0.117	−2.879	0.004	−2.857 to −0.540	1.670
Work experience	1.429	0.652	0.099	2.191	0.029	0.148 to 2.709	2.045
Professional title	−0.111	0.602	−0.008	−0.185	0.854	−1.293 to 1.070	1.871
Employment status	−0.589	0.574	−0.037	−1.026	0.305	−1.717 to 0.538	1.346
Direct patient care	−1.859	1.440	−0.041	−1.291	0.197	−4.686 to 0.968	1.021
Work overtime	−3.795	1.127	−0.109	−3.367	<0.001	−6.008 to −1.583	1.050
Clinical preceptor status	0.350	0.524	0.024	0.669	0.504	−0.677 to 1.378	1.342
Night shifts per month	−0.658	0.459	−0.046	−1.433	0.152	−1.559 to 0.243	1.061
Sleep quality	−3.337	0.660	−0.163	−5.052	<0.001	−4.633 to −2.040	1.054
Perceived patient safety culture (score)	0.157	0.013	0.382	12.029	<0.001	0.131 to 0.183	1.020

Block 1 included six personal and professional background factors. Block 2 added six organizational work environment factors. Block 3 added perceived patient safety culture. Reference categories: age ≤35 years; male; below bachelor’s degree; married; work experience ≤5 years; junior professional title; formal employment; no direct patient care; no work overtime; not a clinical preceptor; ≤10 night shifts per month; and good sleep quality. Perceived patient safety culture was entered as a continuous variable. *R*^2^, coefficient of determination; Δ*R*^2^, change in *R*^2^; SE, standard error; CI, confidence interval; VIF, variance inflation factor.

The hierarchical regression model generally met the diagnostic assumptions, including independence of errors (Durbin–Watson = 1.913), absence of substantial multicollinearity (VIFs = 1.020–2.045), and approximate normality of residuals. The regression diagnostic plots are presented in Supplementary Figure S2. The residuals-versus-predicted plot showed no pronounced funnel-shaped or systematic pattern. Further influence diagnostics and exclusion of the observation with the largest Cook’s distance of 0.029 showed no material change in the unstandardized coefficient for perceived patient safety culture (0.86% change; *p* < 0.001). Block 1 explained 7.7% of the variance in shared governance capacity. Adding the organizational work environment factors in Block 2 explained an additional 4.5% of the variance. Adding perceived patient safety culture in Block 3 explained a further 14.3% of the variance. The final model explained 26.6% of the variance (adjusted *R*^2^ = 0.253, *F* = 20.658, *p* < 0.001).

A higher perceived patient safety culture (*β* = 0.382, *p* < 0.001) and having more than 5 years of work experience (*β* = 0.099, *p* = 0.029) were associated with higher shared governance capacity. In contrast, unmarried status (*β* = −0.117, *p* = 0.004), work overtime (*β* = −0.109, *p* < 0.001), and poor sleep quality (*β* = −0.163, *p* < 0.001) were associated with lower shared governance capacity. Full block-specific regression coefficients are presented in Supplementary Table S3.

A sensitivity analysis using HC3 heteroscedasticity-robust standard errors yielded unchanged coefficient estimates and materially unchanged statistical significance for the principal associations (Supplementary Table S4).

## 4 Discussion

This study found relatively limited capacity for participating in shared governance among nurses in a tertiary hospital in China, with 61.1% of respondents not meeting the qualifying threshold. Compared with previous studies, the proportion observed in this study appeared lower than the proportions reported in some studies from developed countries (6) and comparable to findings from other developing countries, such as Jordan (27). Differences in measurement instruments, organizational contexts, and sample characteristics should be considered when interpreting these comparisons. These findings may suggest substantial room for improvement in nurses’ shared governance capacity in healthcare settings with similar workforce and organizational characteristics.

The positive association between nurses’ perceived patient safety culture and their shared governance capacity was a key finding in this study. Perceived patient safety culture emerged as an important organizational correlate, aligning with previous research emphasizing its association with fostering professional engagement and decision-making participation (28, 29). This association may also be bidirectional. Nurses with stronger shared governance capacity may participate more actively in organizational communication and decision-making processes, which may lead them to report more positive perceptions of patient safety culture (30). Given the associations of overtime work and poor sleep quality with lower shared governance capacity, hospital administrators may need to consider staffing optimization, workload management, and shift scheduling (31). Although the number of night shifts per month was included in the adjusted model, it was not statistically significant. However, its significant univariate association with shared governance capacity suggests that night-shift burden should still be considered in workforce management (32). Such reforms may help support both patient safety culture and nurses’ engagement in shared governance.

This study found that nurses with more than 5 years of work experience reported higher levels of shared governance capacity. In our sample, 74.6% of the participants were 35 years of age or younger, which reflects the overall trend of a younger nursing workforce in China and is consistent with national data showing that over 60% of RNs fall within this age group (33). A substantial proportion of nurses are therefore in the early stages of their careers and have limited clinical and organizational experience. Many of these nurses may not yet have developed a clear professional identity or the competencies required for active participation in governance, which may restrict their engagement in decision-making processes (29, 34).

In addition, China’s professional title system requires nurses to complete specified periods of professional practice or service in the preceding professional title before they become eligible for advancement to a higher professional title (35). Consequently, younger nurses often remain in junior positions with restricted access to formal authority. They are frequently assigned to work night shifts and perform basic care duties, facing heavy workloads while receiving limited institutional support (36). These structural conditions may further be related to fewer opportunities for younger nurses to engage in unit-level governance and may hinder the development of their governance capacity (37).

However, the limited governance capacity observed among younger nurses may not be solely explained by limited experience or junior positions. A possible explanation, supported by previous literature, may lie in the hierarchical structures and cultural dynamics embedded within many nursing organizations (38). A study by Lu et al. (39) suggests that, in most Chinese hospitals, decision-making power is highly centralized in the role of the head nurse. In such settings, staff nurses, particularly those in the early stages of their careers, may have little influence or limited voice in governance-related matters (39). In such authority-driven settings, conforming to nurse managers’ expectations is frequently equated with complying with institutional norms, which may discourage open communication and active involvement (40). A network analysis conducted in five hospitals in Guangdong Province provides related evidence, showing that high cultural power distance was associated with nurses’ reluctance to speak up in front of their superiors, even when they recognize potential issues (39).

This pattern may reflect not only institutional arrangements but also cultural influences described in previous literature. Confucian values, which emphasize respect for hierarchy, collectivism, and deference to authority, may shape interactions and decision-making practices in Chinese healthcare settings (38). Such values may manifest as a strong reliance on rank, seniority, and professional title as proxies for legitimacy and influence (41). Within this cultural framework, younger nurses, regardless of their competence, may defer to more senior colleagues or refrain from contributing out of concern for perceived inadequacy (42). This may contribute to a behavioural pattern in which experience is closely linked to credibility and authority, which may discourage open expression. The combined influence of cultural norms and rigid institutional hierarchies may restrict meaningful participation in governance and may weaken nurses’ sense of belonging and professional autonomy (43).

Overtime work was identified as a significant negative correlate of shared governance capacity. Together with poor sleep quality, this finding suggests that work-related burdens may limit nurses’ available time, energy, and cognitive resources for governance participation. Notably, 96.2% of the nurses in this study reported working overtime, suggesting a substantial workload burden in the clinical setting. Despite improvements in nurse density, with 4 registered nurses per 1,000 people by the end of 2023 (8), China still falls below the GBD-estimated threshold of 7.06 nurses per 1,000 people (9). This persistent workforce shortage may contribute to excessive workloads and may limit opportunities for governance participation or continuing education (44, 45). Previous studies have shown that over 70% of nurses believe they should be compensated for participating in shared governance activities outside of working hours (46). In some settings, nurses’ access to governance-related learning opportunities may be constrained when paid training policies or protected learning time are unavailable. Accordingly, healthcare policymakers may need to consider expanding the nursing workforce and integrating shared governance training into regular working hours or providing appropriate incentives for participation (45).

Poor sleep quality was also negatively associated with shared governance capacity. In this study, 87.7% of the nurses reported having poor sleep quality, and over one-third worked more than 10 night shifts per month. This finding is consistent with those of previous studies indicating that nurses working night shifts are at higher risk for sleep disturbances, which may impair both their clinical performance and governance engagement (47). The increased alertness required during night shifts and limited opportunities for rest and reflection may be related to reduced critical thinking, which is a key component of governance capacity (32). Therefore, night shift management and support systems may need further attention to better support the physical and cognitive wellbeing of frontline staff (48).

## 5 Limitations

Several limitations should be acknowledged. The cross-sectional design limited causal inference. Future longitudinal or interventional studies are needed to examine the direction of the observed associations. Because the principal variables were self-reported, the findings may have been affected by social desirability and reporting biases. The concurrent self-report assessment of perceived patient safety culture and shared governance capacity may also have introduced common-source measurement effects. Sleep quality and working overtime were assessed using single subjective items without fixed recall periods, which may have resulted in some misclassification. Male nurses were underrepresented in the sample, which limited sex-specific comparisons. Future studies should seek to recruit more male nurses where possible. Because this was a single-centre study, the external generalizability of the findings remains constrained. Multicentre and longitudinal collaborative studies are recommended to enhance the broader applicability of findings. Ward- or department-level identifiers were not retained in the analytical dataset. Therefore, possible within-unit clustering could not be evaluated, and the standard errors may have been underestimated. Voluntary participation may have introduced non-response bias, especially among nurses who were absent, heavily burdened, or less engaged with organizational activities. Caution is also needed when interpreting subgroup comparisons and regression estimates involving variables with highly imbalanced categories, as only small proportions of nurses did not work overtime or reported good sleep quality, or did not provide direct patient care. Although the final regression model explained 26.6% of the variance, other unmeasured factors may also contribute to shared governance capacity. These factors may include leadership style, psychological empowerment, organizational climate, and managerial support.

## 6 Conclusion

This study found a relatively low level of shared governance capacity among nurses in a tertiary hospital in China. Shared governance capacity was associated with perceived patient safety culture, work experience, marital status, overtime work, and sleep quality. Healthcare organizations in similar settings may consider strengthening patient safety culture, reducing excessive overtime, optimizing shift schedules, and creating more opportunities for junior nurses to participate in shared decision-making. Future multicentre and longitudinal studies are needed to clarify the direction of these associations and to assess the generalizability of the findings.

## Data Availability

The raw data supporting the conclusions of this article will be made available by the authors, without undue reservation.
