# Supplementary material for: Associations of perceived patient safety culture and work-related characteristics with self-reported shared governance capacity among nurses in a tertiary hospital in China: a cross-sectional study
Source: Front Med (Lausanne). 2026 Aug 19;13:1897058. doi: 10.3389/fmed.2026.1897058 (PMC13533665; doi:10.3389/fmed.2026.1897058)
Supplement: Supplementary file 1 [file Supplementary_file_1.pdf]

Associations of Perceived Patient Safety Culture and Work-Related Characteristics With Self-Reported Shared Governance Capacity Among Nurses in a Tertiary Hospital in China: A Cross-Sectional Study

Supplementary Data (Appendices 1–6)

Contents list

Appendix 1: Figure S1. Flowchart of Participant Recruitment and Questionnaire Inclusion

Appendix 2: Table S1. Operational Definitions of Selected Work-Related Variables

Appendix 3: Table S2. Coding of Dichotomous Variables Used in the Hierarchical Multiple Linear Regression Analysis

Appendix 4: Figure S2. Regression Diagnostic Plots

Appendix 5: Table S3. Full Hierarchical Multiple Linear Regression Analysis

Appendix 6: Table S4. HC3 Heteroscedasticity-Robust Sensitivity Analysis

Appendix 1: Figure S1. Flowchart of Participant Recruitment and Questionnaire Inclusion

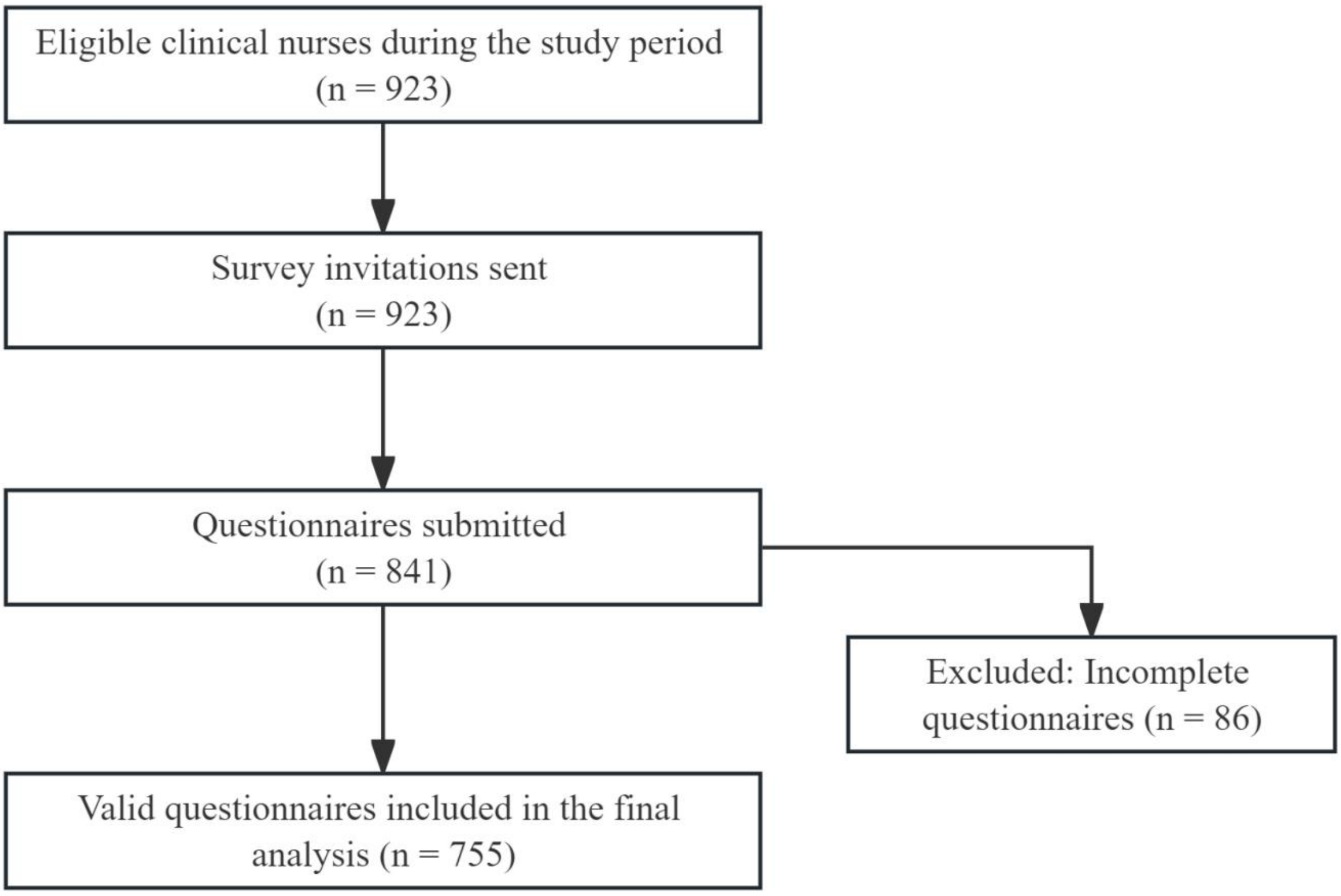

Appendix 2: Table S1. Operational Definitions of Selected Work-Related Variables

Variable	Item wording	Response	Reference period	Additional information
**Work overtime**	Do you usually work beyond your scheduled working hours?	No; Yes	Usual work situation. No fixed recall period	Assessed using a single non-validated item. Overtime frequency and duration were not assessed.
**Sleep quality**	How would you rate your usual sleep quality?	Good; Poor	Usual sleep condition. No fixed recall period	Assessed using a single non-validated self-reported item.
**Work experience**	Total years of nursing work experience	≤5 years; >5 years	Current cumulative work experience	The cutoff was prespecified to distinguish nurses in the earlier stage of clinical practice from those with more established work experience.
**Night shifts per month**	Number of night shifts worked per month	≤10; >10	Typical monthly schedule	More than 10 night shifts per month was used to indicate a relatively high night-shift burden based on routine scheduling in the study hospital.

Note. The cutoffs for work experience and night shifts were prespecified pragmatic study categories rather than validated clinical thresholds.

Appendix 3: Table S2. Coding of Dichotomous Variables Used in the Hierarchical Multiple Linear Regression Analysis

Variable	Variable name	Assignment
Y	Shared governance capacity (score)	
X1	Age	≤ 35 = 0; > 35 = 1
X2	Gender	Male = 0; Female = 1
X3	Education level	Below bachelor's degree = 0; Bachelor's degree and above = 1
X4	Marital status	Married = 0; Unmarried = 1
X5	Work experience	≤ 5 = 0; > 5 = 1
X6	Professional title	Junior = 0; Intermediate or senior = 1
X7	Employment status	Formal employment = 0; Contract-based employment = 1
X8	Direct patient care	No = 0; Yes = 1
X9	Work overtime	No = 0; Yes = 1
X10	Clinical preceptor	No = 0; Yes = 1
X11	Night shifts per month	≤10=0; >10=1
X12	Sleep quality	Good=0; Poor=1

Note. For all dichotomous predictor variables, the category coded as 0 was treated as the reference category.

Appendix 4: Figure S2. Regression Diagnostic Plots

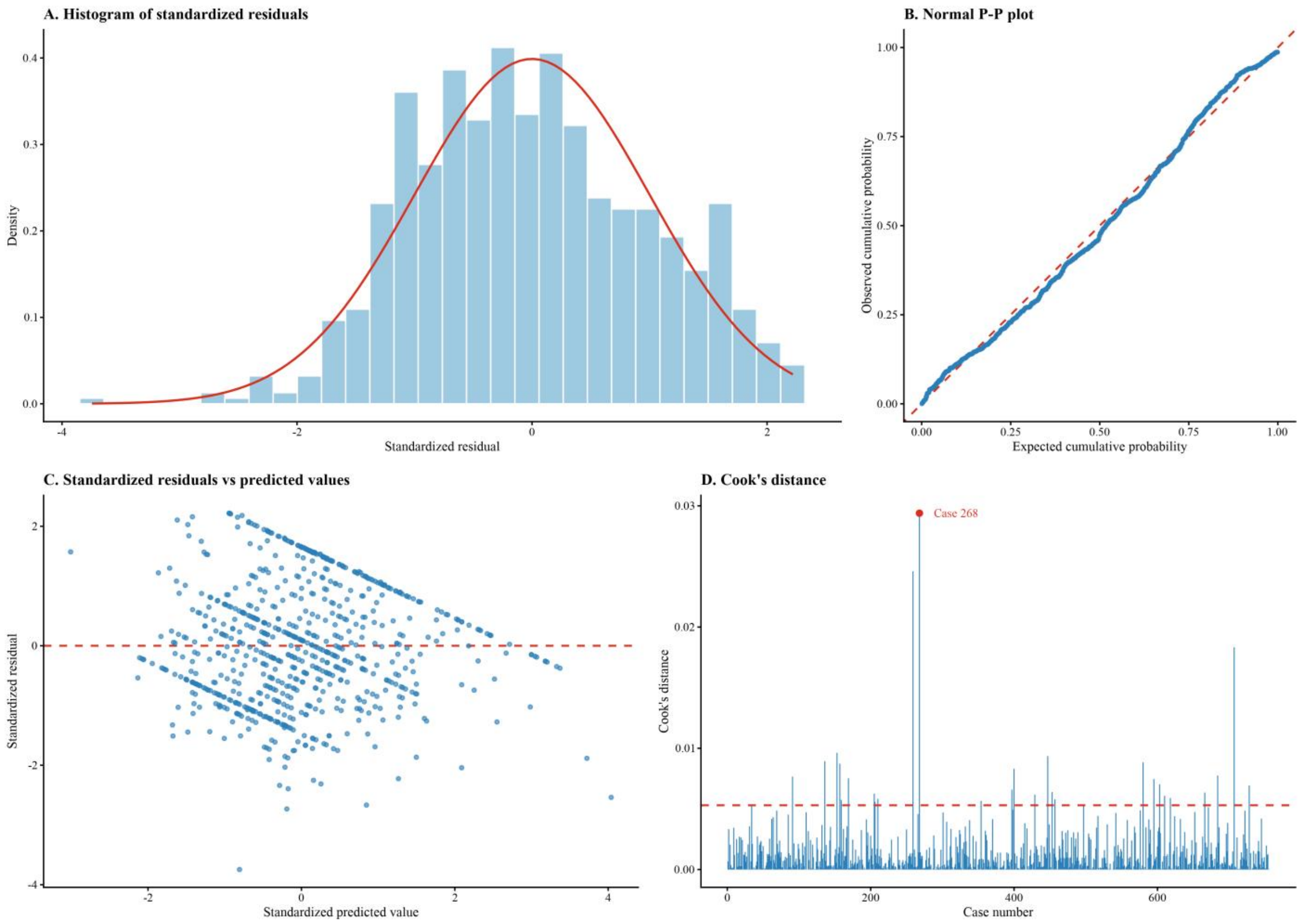

Note. In Panel D, the red dashed line indicates the 4/n Cook's-distance reference line, corresponding to 0.0053 in the present sample.

Appendix 5: Table S3. Full Hierarchical Multiple Linear Regression Analysis of Shared Governance Capacity (n = 755)

Model / independent variable	*B*	*SE*	*β*	*t-value*	*P-value*	*95% CI lower*	*95% CI upper*	*Tolerance*	*VIF*
**Block 1**									
Constant	32.479	1.142		28.444	<0.001	30.237	34.721		
Age	1.277	0.672	0.083	1.900	0.058	-0.042	2.596	0.650	1.538
Gender	2.649	1.023	0.092	2.590	0.010	0.641	4.656	0.970	1.031
Educational level	-0.246	0.568	-0.016	-0.433	0.665	-1.360	0.868	0.851	1.176
Marital status	-1.970	0.655	-0.136	-3.009	0.003	-3.256	-0.685	0.605	1.652
Work experience	1.330	0.716	0.092	1.857	0.064	-0.076	2.736	0.505	1.982
Professional title	0.091	0.635	0.006	0.142	0.887	-1.156	1.337	0.598	1.673
Model Fit	$R^2 = 0.077$, adjusted $R^2 = 0.070$, $\Delta R^2 = 0.077$, F change = 10.436, $P < 0.001$; overall F = 10.436, $P < 0.001$.								
**Block 2**									
Constant	40.311	2.402		16.785	<0.001	35.596	45.026		
Age	0.924	0.684	0.060	1.351	0.177	-0.419	2.266	0.602	1.662
Gender	2.144	1.011	0.075	2.122	0.034	0.160	4.128	0.951	1.051
Educational level	-0.247	0.560	-0.017	-0.441	0.660	-1.347	0.853	0.836	1.196
Marital status	-1.790	0.644	-0.123	-2.777	0.006	-3.055	-0.525	0.599	1.670
Work experience	1.588	0.712	0.110	2.230	0.026	0.190	2.987	0.489	2.044
Professional title	0.008	0.657	0.001	0.012	0.990	-1.283	1.299	0.535	1.871
Employment status	-0.091	0.626	-0.006	-0.145	0.884	-1.320	1.138	0.747	1.339
Direct patient care	-0.981	1.571	-0.022	-0.624	0.533	-4.065	2.104	0.982	1.019
Work overtime	-3.546	1.231	-0.101	-2.880	0.004	-5.963	-1.129	0.953	1.050
Clinical preceptor status	0.095	0.572	0.007	0.167	0.867	-1.027	1.217	0.746	1.340
Night shifts per month	-0.760	0.501	-0.054	-1.517	0.130	-1.745	0.224	0.943	1.060
Sleep quality	-3.198	0.721	-0.156	-4.432	<0.001	-4.614	-1.781	0.949	1.054
Model Fit	$R^2 = 0.123$, adjusted $R^2 = 0.108$, $\Delta R^2 = 0.045$, F change = 6.405, $P < 0.001$; overall F = 8.647, $P < 0.001$.								

Table S3. Continued

Model / independent variable	*B*	*SE*	*β*	*t-value*	*P-value*	*95% CI lower*	*95% CI upper*	*Tolerance*	*VIF*
**Block 3**									
Constant	20.472	2.748		7.449	<0.001	15.077	25.867		
Age	0.579	0.626	0.038	0.925	0.355	-0.650	1.809	0.600	1.666
Gender	1.263	0.928	0.044	1.361	0.174	-0.558	3.085	0.946	1.058
Educational level	-0.242	0.513	-0.016	-0.471	0.638	-1.249	0.765	0.836	1.196
Marital status	-1.699	0.590	-0.117	-2.879	0.004	-2.857	-0.540	0.599	1.670
Work experience	1.429	0.652	0.099	2.191	0.029	0.148	2.709	0.489	2.045
Professional title	-0.111	0.602	-0.008	-0.185	0.854	-1.293	1.070	0.534	1.871
Employment status	-0.589	0.574	-0.037	-1.026	0.305	-1.717	0.538	0.743	1.346
Direct patient care	-1.859	1.440	-0.041	-1.291	0.197	-4.686	0.968	0.979	1.021
Work overtime	-3.795	1.127	-0.109	-3.367	<0.001	-6.008	-1.583	0.952	1.050
Clinical preceptor status	0.350	0.524	0.024	0.669	0.504	-0.677	1.378	0.745	1.342
Night shifts per month	-0.658	0.459	-0.046	-1.433	0.152	-1.559	0.243	0.943	1.061
Sleep quality	-3.337	0.660	-0.163	-5.052	<0.001	-4.633	-2.040	0.948	1.054
Perceived patient safety culture (score)	0.157	0.013	0.382	12.029	<0.001	0.131	0.183	0.981	1.020
Model Fit	R^2 = 0.266, adjusted R^2 = 0.253, ΔR^2 = 0.143, F change = 144.699, P < 0.001; overall F = 20.658, P < 0.001.								

Notes. Block 1 included six personal and professional background factors. Block 2 added six organizational work environment factors. Block 3 added perceived patient safety culture. Reference categories: age ≤35 years; male; below bachelor's degree; married; work experience ≤5 years; junior professional title; formal employment; no direct patient care; no work overtime; not a clinical preceptor; ≤10 night shifts per month; and good sleep quality. Perceived patient safety culture was entered as a continuous variable. R^2, coefficient of determination; ΔR^2, change in R^2; SE, standard error; CI, confidence interval; VIF, variance inflation factor.

Appendix 6: Table S4. HC3 Heteroscedasticity-Robust Sensitivity Analysis of the Final Regression Model (n = 755)

Predictor	B	HC3 robust SE	HC3 t	HC3 P	HC3 95% CI Lower	HC3 95% CI Upper
Constant	20.472	2.620	7.815	< 0.001	15.329	25.615
Age	0.579	0.636	0.911	0.363	-0.669	1.828
Gender	1.263	0.774	1.633	0.103	-0.255	2.782
Education level	-0.242	0.489	-0.494	0.621	-1.202	0.718
Marital status	-1.699	0.563	-3.016	0.003	-2.804	-0.593
Work experience	1.429	0.642	2.225	0.026	0.168	2.689
Professional title	-0.111	0.598	-0.186	0.853	-1.285	1.063
Employment status	-0.589	0.564	-1.044	0.297	-1.697	0.519
Direct patient care	-1.859	1.638	-1.135	0.257	-5.075	1.357
Work overtime	-3.795	1.056	-3.594	< 0.001	-5.868	-1.722
Clinical preceptor	0.350	0.513	0.683	0.495	-0.656	1.357
Night shifts per month	-0.658	0.460	-1.429	0.154	-1.561	0.246
Sleep quality	-3.337	0.663	-5.033	< 0.001	-4.638	-2.035
Perceived patient safety culture (score)	0.157	0.011	13.662	< 0.001	0.134	0.180

Note. HC3 robust estimation adjusted the standard errors and corresponding inferential statistics without changing the coefficient estimates. The direction and statistical significance of all principal associations remained unchanged.
